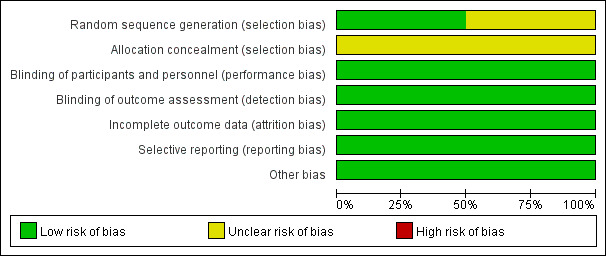# Incretin-based drugs decrease the incidence of prostate cancer in type 2 diabetics: A pooling-up analysis

**DOI:** 10.1097/MD.0000000000038018

**Published:** 2024-05-17

**Authors:** Yuxiang Lin, Guangyong Xu, Liangyu Li, Jingyi Xiang, Lingyun Zhai

**Affiliations:** a Department of Urology, The Second Affiliated Hospital of Chongqing Medical University, Chongqing, China; b Department of Infectious Diseases, Key Laboratory of Molecular Biology for Infectious Diseases (Ministry of Education), Institute for Viral Hepatitis, The Second Affiliated Hospital of Chongqing Medical University, Chongqing, China.

**Keywords:** based drugs, DPP-4, GLP-1, incretin, prostate cancer

## Abstract

Incretin-based drugs, a class of Antidiabetic medications (ADMs) used in the treatment of type 2 diabetes, may affect the incidence of prostate cancer (PCa). But real-world evidence for this possible effect is lacking. Therefore, the aim of this study is to assess the effect of incretin-based drugs on the incidence of PCa, including glucagon-like peptide-1 (GLP-1) receptor agonists and dipeptidyl peptidase-4 (DPP-4) inhibitors. We searched PubMed, Embase, and Cochrane Library databases for eligible studies through September 2023. Two independent reviewers performed screening and data extraction. We used the Cochrane Handbook for Systematic Reviews and the Newcastle-Ottawa Scale (NOS) to assess the quality of included randomized controlled trials (RCTs) and cohort studies. We did a meta-analysis of available trial data to calculate overall risk ratios (RRs) for PCa. A total of 1238 articles were identified in our search. After screening for eligibility, 7 high-quality studies met the criteria for meta-analysis, including 2 RCTs and 5 cohort studies, with a total of 1165,738 patients. Compared with the control group, we found that incretin-based drugs reduced the relative risk of PCa by 35% (95% confidence interval (CI), 0.17–0.49; *P* = .0006). In subgroup analysis, the RR values for GLP-1 receptor agonists and DPP-4 inhibitors were 62% (95% CI, 0.45–0.85; *P* = .003) and 72% (95% CI, 0.46–1.12; *P* = .14), respectively. Incretin-based drugs are associated with lower incidence of prostate cancer and may have a preventive effect on prostate cancer in patients with type 2 diabetes.

## 1. Introduction

In recent years, the relationship between Antidiabetic medications (ADMs) and various cancer risks in type 2 diabetes has received much attention.^[1]^ Multiple studies have shown that patients with type 2 diabetes who use ADMs have either an increased or decreased risk of colorectal, liver, thyroid, pancreatic, endometrial, and prostate cancer (PCa).^[2–7]^ Incretin-based drugs, the new types of ADMs, such as saxagliptin and liraglutide can significantly lower blood glucose. Also, they reduce body weight and blood pressure, showing the potential to slow the progression of diabetes and reduce the cardiovascular complications of diabetes.^[8,9]^ Thus, incretin-based drugs have likewise received some attention for their association with PCa risk.^[10]^

Several basic studies have shown that incretin-based drugs can inhibit the growth of prostate cancer cells in in vitro assays. Experimental study showed that exendin-4, one of the GLP-1 receptor agonists, significantly reduces tumor volume when LNCap cells are transplanted into thymopathic mice.^[11]^ This is due to the fact that GLP-1R mRNA was abundantly expressed in LNCap cells, and exendin-4 reduces the proliferation of prostate cancer cells through the activation of the GLP-1 receptor, thereby inhibiting ERK-MAPK. A recent study showed that DPP-4 inhibitors were thought to be primarily responsible for modulating the activity of a range of peptides, such as GLP-1 and glucose-dependent insulinotropic polypeptide, thereby inhibiting or promoting the risk of cancers, including PCa.^[12]^ Moreover, Wesley et al found that DPP-4 inhibits the malignant phenotype of prostate cancer cells by blocking bFGF signaling pathway.^[13]^ However, the role between incretin-based drugs and prostate cancer in real populations has not yet been fully demonstrated.

In recent years, multicenter RCTs such as the LEADER clinical trial (Liraglutide Effect and Action in Diabetes: Evaluation of Cardiovascular Outcome Results) and the SAVOR-TIMI 53 clinical trial (Saxagliptin Assessment of Vascular Outcomes Recorded in Patients with Diabetes Mellitus-Thrombolysis in Myocardial Infarction) published the tumor-related adverse event outcomes for incretin-based drugs.^[14,15]^ On the other hand, there are also recently published cohort studies that have studied the relationship between incretin-based drugs and PCa.^[16–20]^ However, there are still controversies that remain.

The aim of the present meta-analysis was to collect all available evidence from high-quality RCTs and cohort studies in order to evaluate the effect of incretin-based therapy on the incidence of PCa.

## 2. Materials and methods

### 2.1. Search strategy and selection criteria

This study was planned and reported in accordance with the Preferred Reporting Items for Systematic Reviews and Meta-Analyses (PRISMA) guidelines.^[21]^ And it was registered on PROSPERO. In this meta-analysis, we searched PubMed, Embase, and Cochrane Library (from inception to September 2023) for all relevant studies. The search terms used were given below: “dipeptidyl peptidase iv inhibitor,” “DPP-4,” “DPP4,” “alogliptin,” “sitagliptin,” “saxagliptin,” “linagliptin,” “vildagliptin,” “glucagon like peptide 1 receptor agonists,” “GLP-1,” “GLP1,” “albiglutide,” “dulaglutide,” “exenatide,” “liraglutide,” “semaglutide,” “lixisenatide,” “oral semaglutide” combined with “cancer.” Only English-language articles were included.

Studies were included if they satisfied all of the following criteria: the study population consisted of patients with type 2 diabetes; the study evaluated the risk ratio of PCa incidence with incretin-based therapy compared to the control group; and absolute numbers for the total number of male patients in the experimental group, the number of events, the total number of male patients in the control group, and the number of events outcome could be adequately extracted.

If studies met one of the following criteria, they were excluded: the study was a review or meta-analysis; the reported populations had substantial overlap; the total samples in each group were <100; the antidiabetic medications regimen in the experimental and/or control groups was not monotherapy, or monotherapy data could not be segregated.

### 2.2. Data extraction and quality assessment

Two authors (Y.L. and L.Z.) independently screened all titles and abstracts retrieved from the databases. If the article was deemed eligible by either author, the full-text of the article was read by both authors to determine whether the study should be included; there was complete agreement regarding the included articles. The 2 authors then independently extracted all data from all included studies. Discrepancies were resolved by consensus. Studies that did not fulfill all of the inclusion criteria were excluded.

First author name, year of publication, country, mean age, study design, median follow-up duration, specific incretin-based and control drugs, the total number of men, the number of events, relative risk, and their 95% CIs were extracted from the retrieved literature. The above data are shown in Table S1, http://links.lww.com/MD/M338.

To evaluate the methodological quality of the included cohort studies, the Newcastle-Ottawa Scale (NOS) was used (maximum stars of 9).^[22,23]^ And the Cochrane Handbook for Systematic Reviews was used to assess the methodological quality of the included RCTs.^[24,25]^ Each study was independently evaluated by 2 authors (Y.L. and G.X.). Discrepancies were resolved by discussion or, if disagreement still existed, by third-author (L.Z.) arbitration.

### 2.3. Statistical analysis

We performed a meta-analysis using RevMan software (Review Manager, version 5.3) and calculated the statistic heterogeneity through I^2^ and *P* value.^[26–29]^ When I^2^ < 50% and *P* > .1, we ignored heterogeneity and chose the fixed-effects model; when I^2^ > 50% and/or *P* < .1, we chose the random-effects model and explored the source of heterogeneity through subgroup analysis and sensitivity analysis. The experimental group was divided into 2 sub-treatment groups, DPP-4 inhibitors and GLP-1 receptor agonists, depending on the intervention. The risk ratio was calculated as the effect measure to compare the incidence of PCa in patients with type 2 diabetes between the incretin-based treatment group and the control group. Effect size and a 95% CI were calculated and displayed as forest plots. Potential publication bias was determined using the funnel plot.^[29,30]^

## 3. Results

### 3.1. Eligible studies and quality assessment

**Figure 1.**
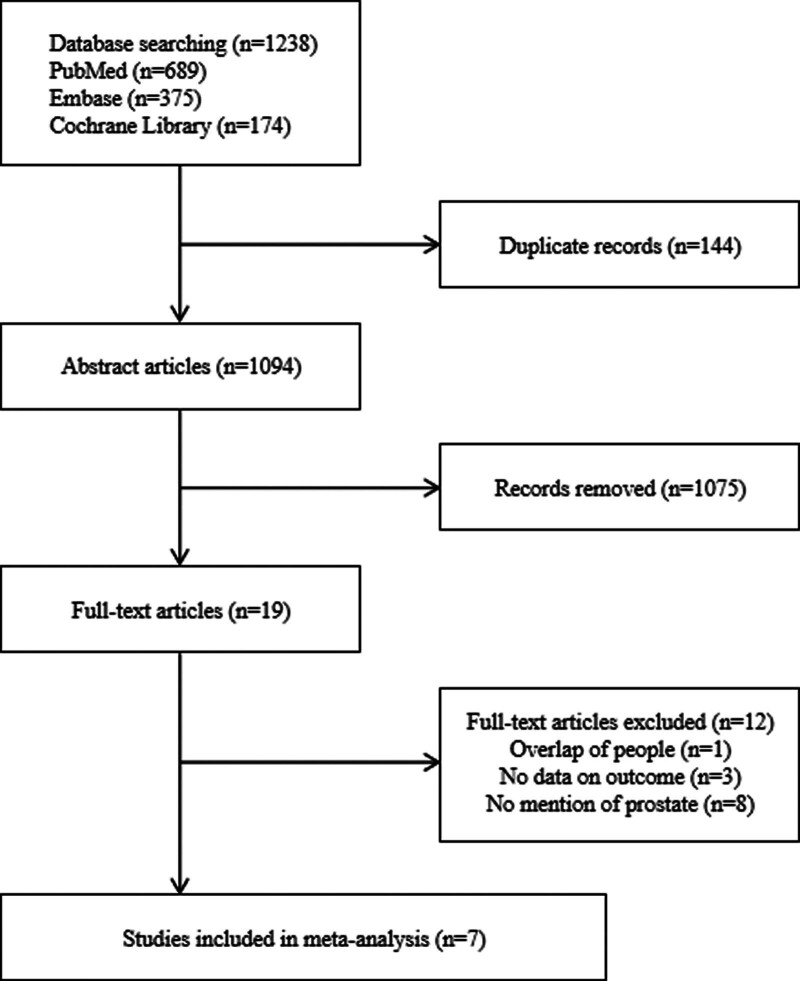
Study selection.

A total of 1238 articles were initially retrieved. After eliminating duplicate articles and reviewing the titles and abstracts, we excluded 1219. After a full-text review, 12 more were excluded, leaving 7 studies remaining to be included in the final analysis (Fig. 1).

The characteristics of the included studies are shown in Table S1, http://links.lww.com/MD/M338. The 7 articles selected included 2 RCTs^[14,15]^ and 5 cohort studies,^[16–20]^ all of which were population-based studies. In the DPP-4 inhibitor subgroup, 3 cohort studies and 1 RCT were included, while the composition of studies in the GLP-1 receptor agonists subgroup was also 3 cohort studies and 1 RCT. One of the 5 cohort studies examined both DPP-4 inhibitors and GLP-1 receptor agonists. Among the included studies, encompassing a total of 1165,738 patients, 3 studies were conducted in Europe, 1 study from the USA, 1 study from Asia, and 2 studies were multicenter RCTs.

The methodological quality was evaluated for the included RCTs (Figure S1, http://links.lww.com/MD/M336). It can be seen that both of the articles had only a low-risk of bias, which got 5 and above scores and were high-quality. Figure S2, http://links.lww.com/MD/M337 showed the summary graph for the risk of bias in the methodological quality assessment. The mean NOS score for the 5 cohort studies included in the meta-analysis was 8.4, including 3 studies with 8 scores and 2 studies with 9 scores (Table S2, http://links.lww.com/MD/M339). Thus, we did not exclude any RCT or cohort study from the analysis. A complete summary of the cohort studies quality assessment was provided in the supplemental material.

### 3.2. Incretin-based drugs and risk of prostate cancer

**Figure 2.**
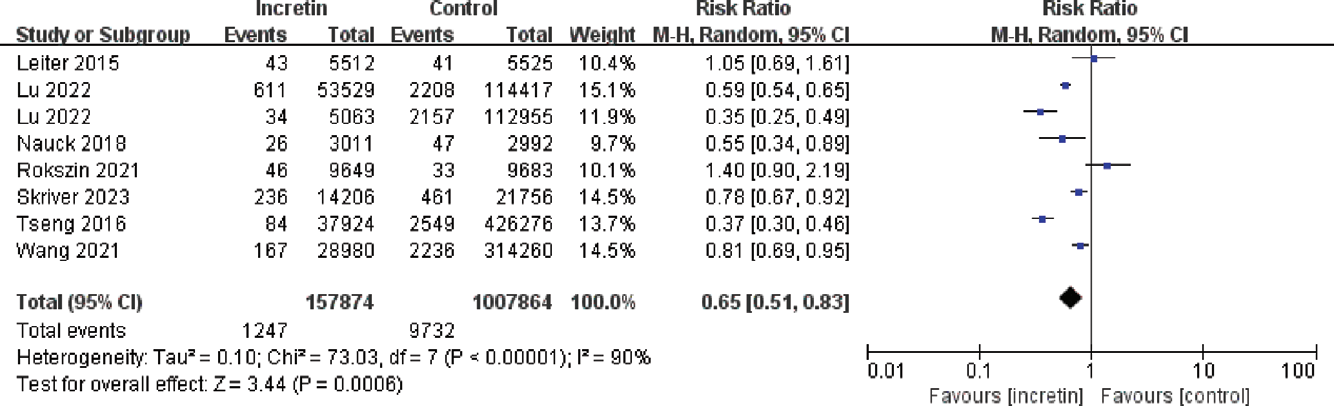
Forest plot for the relationship between incretin-based drugs and the incidence of prostate cancer.

Across all study designs (RCT, cohort studies), there were 157,874 total male patients and 1247 events in the experimental group, while 1,007,864 total male patients and 9732 events in the control group. What more, the RR value was 0.65 (95% CI, 0.51–0.83) Z = 3.44, *P* = .0006 < 0.05. It meant that patients with type 2 diabetes treated with incretin-based drugs had an average 35% lower relative risk of PCa events compared to controls (Fig. 2). The result was statistically significant.

### 3.3. Heterogeneity

As shown above (Fig. 2), the 7 studies in this meta-analysis were examined for heterogeneity with I^2^ = 90% > 50% and *P* < .00001, suggesting that the heterogeneity among the papers selected for this study was statistically significant and a search for heterogeneity was necessary.

A sensitivity analysis of the study did not reveal any literature with a large effect on heterogeneity. The subgroup analysis is shown below.

#### 3.3.1. DPP-4 inhibitor subgroup analysis

**Figure 3.**
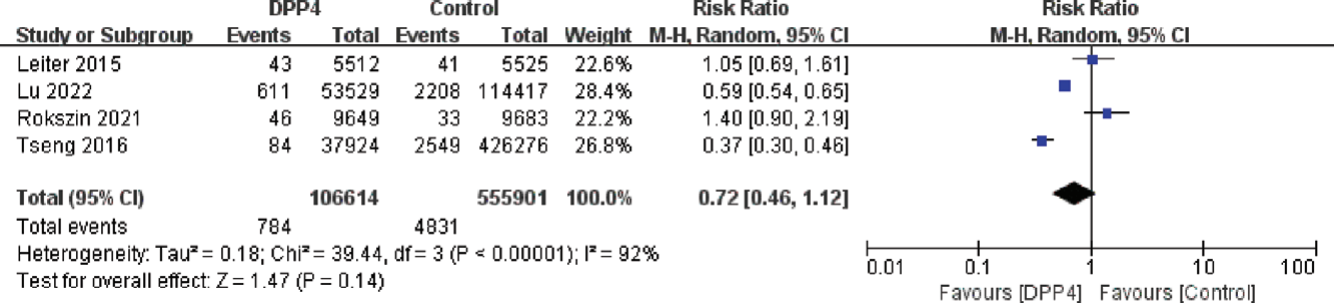
Forest plot for the relationship between dipeptidyl peptidase-4 and the incidence of prostate cancer in subgroup analysis.

A total of 4 studies were entered into the DPP-4 inhibitors group. A test for heterogeneity was performed and showed a large heterogeneity of studies (I^2^ = 92% > 50% and *P* < .00001). The random-effects model was chosen to create a forest plot after combining the effect sizes. Figure 3 showed that the RR value was 72% (95% CI, 0.46–1.12), and there was a tendency for the DPP-4 inhibitor group to have a lower incidence of PCa when compared with the control group, but the difference was not significant (Z = 1.47, *P* = .14).

A sensitivity analysis revealed that Rockszin et al had a large effect on heterogeneity.^[17]^ Compare with other studies, the drug of control group in this study was SGLT2i, which was a novel drug developed recently. Removal of this study changed the results of this subgroup, the final result suggested that the DPP-4 inhibitors reduced the relative risk of PCa compared to the control group, which was statistically significant (Z = 2.41, *P* = .02 < 0.05).

#### 3.3.2. GLP-1 receptor agonists subgroup analysis

**Figure 4.**
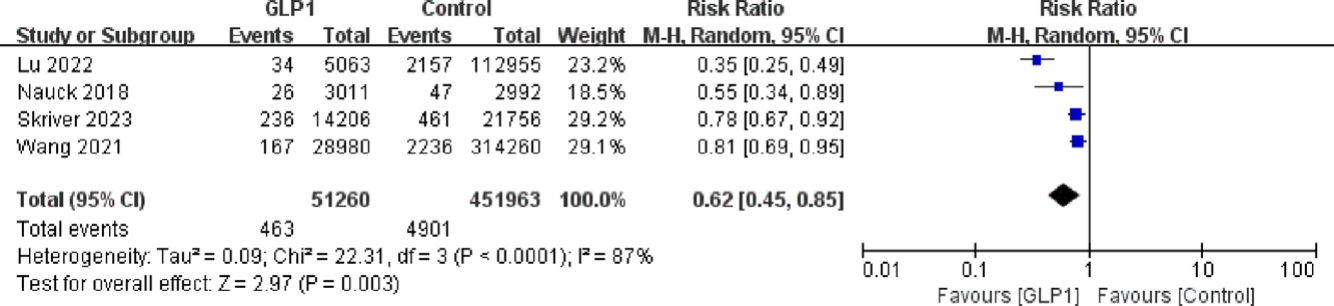
Forest plot for the relationship between glucagon-like peptide-1 and the incidence of prostate cancer in subgroup analysis.

A heterogeneity test was performed on the 4 studies in the GLP-1 receptor agonists group, which similarly showed a large heterogeneity of studies (I^2^ = 87% > 50% and *P* < .0001). Similar to the DPP4 inhibitor subgroup, we chose a random-effects model for the forest plot after combining effect sizes. Figure 4 showed that the RR value was 62% (95% CI, 0.45–0.85), and the difference between GLP-1 receptor agonists and controls was statistically significant (Z = 2.97, *P* = .003). That meant GLP-1 receptor agonists reduced the relative risk of PCa compared to the control group.

A sensitivity analysis revealed that Lu et al had a large effect on heterogeneity.^[18]^ The heterogeneity test after removing this study suggested that there was no heterogeneity in the remaining 3 studies (I^2^ = 13% < 50% and *P* = .32 > 0.1). After excluding this study, a meta-analysis was performed on the remaining studies using the fixed-effects model. And the result suggested the GLP-1 receptor agonists reduced the relative risk of PCa compared to the control group, which was still statistically significant (Z = 4.47, *P* < .00001).

### 3.4. Publication bias

**Figure 5.**
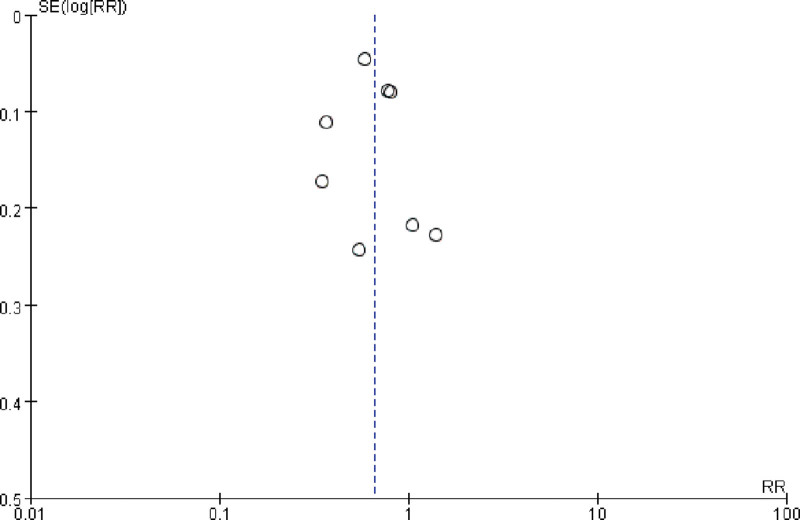
Funnel plot for the association of Incretin-based drugs administration and risk of prostate cancer.

As presented in Figure 5, the funnel plot was symmetrically distributed, indicating that publication bias did not exist.

## 4. Discussion

This meta-analysis summarized the available data on the relationship between incretin-based drugs administration and the risk of PCa in patients with type 2 diabetes.

GLP-1 exerts various effects through its receptors and/or different signal transduction pathways. GLP-1 receptor mRNA is abundantly expressed in LNCap, an androgen-independent human PCa cell line. Li et al found that exenatide and liraglutide inhibited proliferation and induced apoptosis in LNCap.^[31]^ As we mentioned earlier, Nomiyama et al found that exendin-4, as 1 of GLP-1 receptor agonists reduced tumor volume primarily by affecting LNCap cells.^[11]^

The risk relationship between GLP-1 receptor agonists and other malignant tumors have been studied. Two animal experiments with mice found that GLP-1 receptor agonists increased the risk of pancreatic, and thyroid cancers. But they are only a preclinical study, not based on human or other primates.^[32,33]^ Moreover, several meta-analysis suggested that GLP-1 receptor agonists did not associate with the incidence of pancreatic, thyroid, and breast cancers.^[34–38]^

Our study demonstrated that GLP-1 receptor agonists reduced the relative risk of PCa. The result remained significant even after excluding Lu study.^[18]^ It revealed that GLP-1 receptor agonists might have potential preventive effect on prostate cancer compared to the control group.

Another aspect, DPP-4 inhibitors limited the degradation of GLP-1 and glucose-dependent insulinotropic polypeptide to exert effects similar to GLP-1 receptor agonists.^[12]^ Besides, Yang et al found that circular RNA-DPP-4 serves an oncogenic role in prostate cancer progression through regulating miR-195/cyclin D1 axis.^[39]^

Previous meta-analysis showed no significant correlation between DPP-4 inhibitors and some malignancies, including pancreatic and thyroid cancers.^[38,40–42]^ In the present meta-analysis, the result suggested that DDP-4 inhibitors had a tendency to reduce the incidence of PCa. The sensitivity analysis suggested that cohort studies of Rockszin et al have a large impact on the outcome of our DPP-4 inhibitors subgroup.^[17]^ We analyzed that this could be related to the drugs called SGLT-2 inhibitors in the control group. A meta-analysis by Benedetti et al indicated that SGLT-2 inhibitors were significantly associated with a reduced overall cancer risk compared with placebo (RR = 0.35, CI 0.33–0.37).^[43]^ In the meantime, there was no significant difference in the preventive effect of SGLT-2 inhibitors versus DPP-4 inhibitors on prostate cancer in this cohort study. This certainly supported our assumption that DPP-4 inhibitors might have a preventive effect on cancer. Furthermore, when we removed Rockszin study, there was a statistically significant reduction in the incidence of PCa with DPP-4 inhibitors.^[17]^ So we could still consider that DPP4 inhibitors might have potential preventive effect on PCa.

There are several limitations in this study. Firstly, most of the included studies lack long-term follow-up (<5 years). This represented a subset of patients who had a prostate cancer event during subsequent ADMs therapy but were not included in the event group, thus creating bias. Secondly, only 7 studies were included in this meta-analysis, and differences of drug types in control group might have influenced the final RR values and other relevant results of this meta-analysis. However, due to the limitations in the number of included studies, we were unable to do further analysis. Thirdly, although the sample sizes of cohort studies were all large, they were only representative of populations in selected countries. At the same time, the cohort studies were all retrospective, not prospective, which might result in bias.

## 5. Conclusion

Incretin-based drugs are associated with lower incidence of prostate cancer and may have a preventive effect on prostate cancer in patients with type 2 diabetes.

## Acknowledgments

All authors have no conflicts of interest to disclose. And there is no funding for this article.

## Author contributions

**Conceptualization:** Yuxiang Lin, Guangyong Xu, Lingyun Zhai.

**Data curation:** Yuxiang Lin, Guangyong Xu, Liangyu Li, Jingyi Xiang, Lingyun Zhai.

**Formal analysis:** Yuxiang Lin, Guangyong Xu, Liangyu Li, Jingyi Xiang.

**Methodology:** Yuxiang Lin, Guangyong Xu, Lingyun Zhai.

**Software:** Yuxiang Lin, Guangyong Xu.

**Supervision:** Lingyun Zhai.

**Validation:** Lingyun Zhai.

**Visualization:** Yuxiang Lin, Guangyong Xu.

**Writing – original draft:** Yuxiang Lin, Guangyong Xu.

**Writing – review & editing:** Lingyun Zhai.

## Supplementary Material

**Figure:**
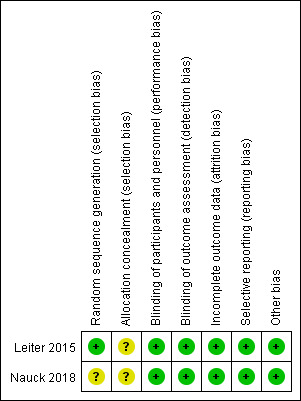


**Figure:**